# Genetic Variations of the Vitamin D Metabolic Pathway and COVID-19 Susceptibility and Severity: Current Understanding and Existing Evidence

**DOI:** 10.3390/biomedicines11020400

**Published:** 2023-01-29

**Authors:** Nipith Charoenngam, Aunchalee Jaroenlapnopparat, Sofia K. Mettler, Ashna Grover

**Affiliations:** 1 Department of Medicine, Mount Auburn Hospital, Harvard Medical School, Cambridge, MA 02138, USA; 2 Department of Medicine, Faculty of Medicine Siriraj Hospital, Mahidol University, Bangkok 73170, Thailand

**Keywords:** vitamin D, 25-hydroxyvitamin D, vitamin D receptor, vitamin D-binding protein, DBP, GC protein, COVID-19, SARS-CoV-2, polymorphism, genetic variation

## Abstract

The immunomodulatory and metabolic effects of vitamin D receptor (VDR) activation have been considered beneficial in mitigating the susceptibility and severity of COVID-19 infection. Furthermore, vitamin D-binding protein (DBP) has pleiotropic effects on the immune system that may influence inflammation associated with COVID-19. Multiple observational studies have demonstrated an association between low levels of serum 25-hydroxyvitamin D and risk and the severity of COVID-19 infection. However, the impact of vitamin D supplementation as an adjunctive treatment for COVID-19 based on evidence from randomized clinical trials is unclear. Equally important is that certain variations of the genes involved in the vitamin D metabolic pathway have been shown to affect immune function and linked with various clinical outcomes, including cardio-metabolic disorders, autoimmune diseases, infections, and cancers. This indicates inter-individual difference in body response to vitamin D. There is also emerging evidence that common polymorphisms of these genes may influence the susceptibility and severity of COVID-19, although the confidence of these findings is limited by a small number of studies and participants. Further studies are needed to address the potential role of VDR activation and DBP in the pathophysiology of COVID-19 which take into account the genetic variations of vitamin D metabolic pathway.

## 1. Introduction

Severe acute respiratory syndrome coronavirus 2 (SARS-CoV-2) is a novel strain of coronavirus that causes coronavirus disease 2019 (COVID-19). It quickly spread worldwide resulting in a pandemic due to its high transmissibility and infectivity [1,2]. While most patients with COVID-19 develop mild respiratory symptoms, a significant proportion of patients develop severe illness resulting in multiple organ failure and death [3,4]. Factors influencing the risk of developing severe COVID-19 include advanced age, smoking, and the presence of underlying comorbidities such as immunocompromised state, cardio-metabolic disorders, chronic kidney disease, and chronic lung disease [5]. 

Multiple studies have suggested that vitamin D deficiency may be associated with increased susceptibility to COVID-19 infection and risks of severe illness [6,7,8,9,10,11]. This indicates the potential therapeutic and preventive role of vitamin D in COVID-19, which not only is responsible for calcium and phosphate homeostasis, but also has biological actions on various tissues that express the vitamin D receptor (VDR) [12,13,14]. Interestingly, there is mounting evidence that genetic variations of proteins involved in vitamin D metabolic pathway can affect tissue response to vitamin D and thus may influence the risks and severity of multiple chronic diseases, including COVID-19. In this article we dissect the complexity of the vitamin D metabolic pathway and outline the skeletal and non-skeletal actions of vitamin D. We review the relationship between vitamin D and COVID-19 infection. We comprehensively assess the recent evidence on genetic variations of vitamin D metabolic pathway in association with various clinical outcomes and COVID-19 and also introduce the concept of individual responsiveness to vitamin D. Finally, we discuss how certain genetic polymorphisms in vitamin D-related genes could impact immune function. 

## 2. Sources, Synthesis, and Metabolism of Vitamin D

Humans obtain vitamin D mainly from diet, supplements, and sunlight exposure. Vitamin D_2_, or ergocalciferol, is derived from the ingestion of yeasts and mushrooms [13,14]. Vitamin D_3_, or cholecalciferol, is endogenously synthesized from 7-dehydrocholesterol in the skin that is exposed to the UVB radiation and exogenously derived from animal products (i.e., cod liver oil, oily fish). Both vitamin D_2_ and D_3_ also exist in the form of supplements and fortification in dietary products, such as milk, cooking oil, and orange juice [13,14].

Once vitamin D (D_2_ and D_3_) enters the circulation it is either distributed and stored in the muscle and adipose tissue or metabolized by the enzyme 25-hydroxylase (mainly encoded by the *CYP2R1* gene) in the liver into 25-hydroxyvitamin D [25(OH)D]. 25(OH)D is the major circulating form of vitamin D clinically measured to reflect vitamin D status in the body. 25(OH)D is further metabolized in the kidney by the enzyme 1α-hydroxylase (encoded by the *CYP27B1* gene) into 1,25-dihydroxyvitamin D [1,25(OH)_2_D], which is the biologically active form of vitamin D [13,14]. Both the circulating 25(OH)D and 1,25(OH)_2_D bind with the vitamin D binding protein (DBP, encoded by the *GC* gene, produced by the liver). For most cells, only the unbound 25(OH)D and 1,25(OH)_2_D enter the cells, but in some organs (i.e., kidney, parathyroid glands and placenta), DBP contributes to the transport of 25(OH)D into the cell via a megalin/cubilin complex [15]. 

1,25(OH)_2_D interacts with the intracellular nuclear VDR (encoded by the *VDR* gene) that forms a heterodimer complex with the retinoid X receptor (RXR) in the target cells. The activated VDR-RXR heterodimer complex, in turn, alters the expression of its target genes by interacting with the vitamin D-response elements (VDREs) in the target genes [16,17]. The 1,25(OH)_2_D-VDR-RXR-VDRE interaction also regulates 1,25(OH)_2_D level by inducing its destruction by upregulating the expression of the gene *CYP24A1*, encoding the enzyme 25-hydroxyvitamin D-24-hydroxylase that metabolizes 1,25(OH)_2_D and 25(OH)D into inactive carboxylic acids [18]. In addition, the enzyme 1α-hydroxylase, responsible for conversion of 25(OH)D into 1,25(OH)_2_D in the kidney is enhanced by the action of parathyroid hormone (PTH) and inhibited by the actions of 1,25(OH)_2_D and fibroblast growth factor-23 (FGF23) [13,14,19]. 

**Figure 1:**
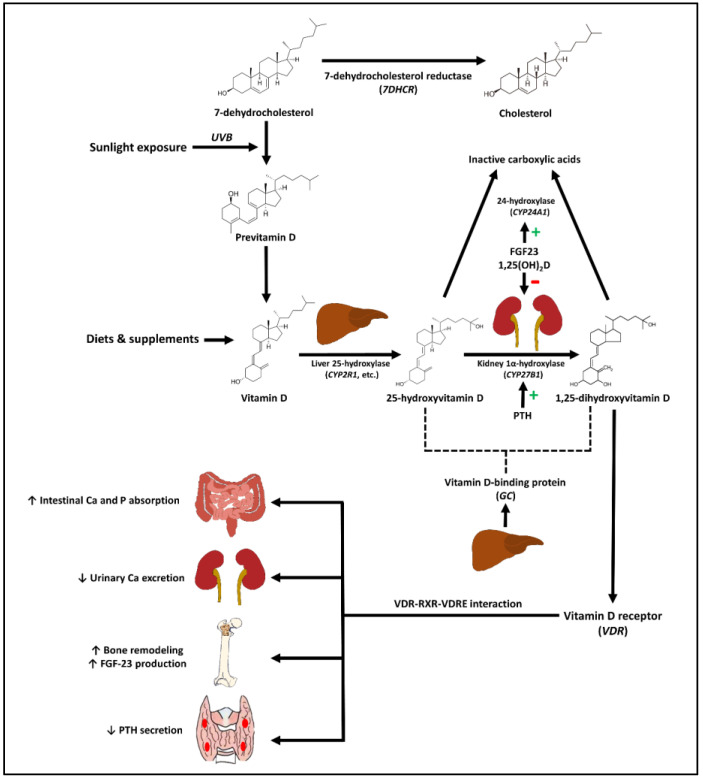
Schematic representation of vitamin D metabolic pathway and the effects of vitamin D on calcium and phosphate homeostasis. Abbreviations: Ca: Calcium; FGF-23; Fibroblast growth factor-23; P: Phosphate; PTH: Parathyroid hormone; *UVB*: Ultraviolet-B radiation.

VDR is widely expressed in various types of tissues including the skin, skeletal muscles, adipose tissues, endocrine pancreas, innate and adaptive immune cells, blood vessels, brain, breast, and placenta [13,14]. Although the kidneys are the main site of production of circulating 1,25(OH)_2_D, *CYP27B1* is expressed in many other types of cells, such as immune cells, parathyroid, breast, microglia, colon, and keratinocytes [6,20]. It is therefore speculated that 25(OH)D may be converted locally in these tissues to 1,25(OH)_2_D to exert its tissue-specific actions in a paracrine or autocrine manner. However, the fate of vitamin D metabolites in different target tissues needs more investigation. A schematic representation of the synthesis and metabolism of vitamin D is shown in Figure 1. 

## 3. Skeletal and Non-Skeletal Effects of Vitamin D 

Activation of VDR affects calcium and phosphate homeostasis mainly by promoting intestinal calcium and phosphate absorption and renal tubular calcium reabsorption. VDR activation increases bone resorption by inducing the receptor activator of nuclear factor kappa B (RANK)-mediated osteoclast differentiation and activation. In addition, 1,25(OH)_2_D directly inhibits PTH production and induces FGF23 production by osteocytes as a part of negative feedback loops to maintain serum calcium and phosphate concentrations within the physiologic ranges [13,14,19] (Figure 1). 

**Figure 2:**
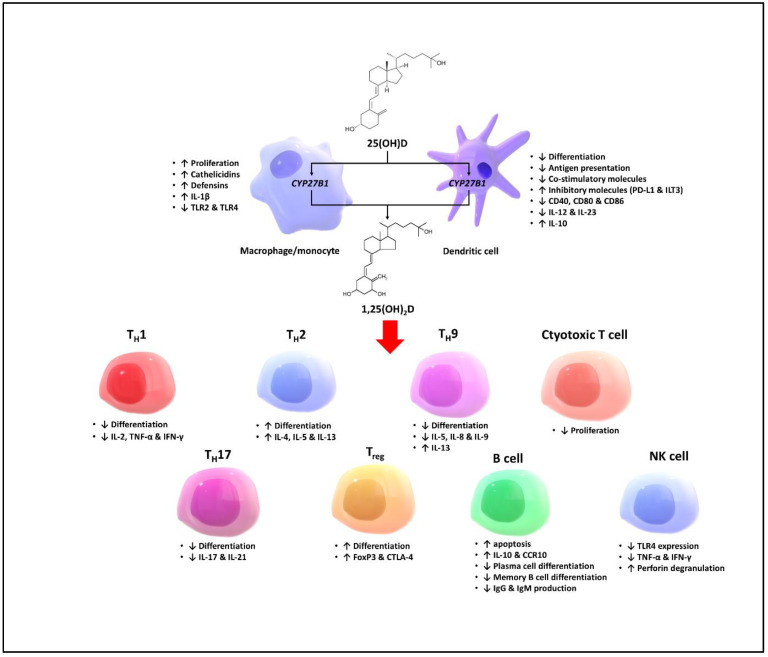
Effects of vitamin D on different cell types of the innate and adaptive immune systems. Abbreviations: 1,25(OH)_2_D: 1,25-dihydroxyvitamin D; 25(OH)D: 25-hydroxyvitamin D; CCR10: C-C chemokine receptor type 10; CTLA-4: cytotoxic T-lymphocyte-associated protein 4; CYP27B1: Cytochrome P450 family 27 subfamily B member 1; FoxP3: Foxhead box P3; IFN-γ: Interferon-γ; IgG: Immunoglobulin G; IgM: Immunoglobulin M; IL-1β; Interleukin-1β; IL-2: Interleukin-2; IL-4: Interleukin-4; IL-5: Interleukin-5; IL-8: Interleukin-8; IL-9: Interleukin-9; IL-10: Interleukin-10; IL-13: Interleukin-13; IL-17: Interleukin-17; IL-21: Interleukin-21; ILT3: Immunoglobulin-like transcript-3; NK: Natural killer; PD-L1: Programmed death-ligand 1; T_H_1; T helper 1; T_H_2: T helper 2; T_H_9: T helper 9; T_H_17: T helper 17; T_reg_: Regulatory T cell; TNF-α; Tumer necrosis factor-α; TLR2: Toll-like receptor 2; TLR4: Toll-like receptor 4 [21].

Vitamin D is also known as an immunomodulatory agent that regulates the innate and adaptive immune systems [12,21,22,23]. Activated macrophages express the enzyme 1α-hydroxylase (CYP27B1) that metabolizes 25(OH)D into 1,25(OH)_2_D, a process which is not regulated by PTH, unlike in the kidneys. 1,25(OH)_2_D acts in a paracrine and autocrine manner to promote the production of the endogenous antimicrobial peptides (i.e., cathelicidins and defensins) by the macrophages [24,25,26]. 1,25(OH)_2_D affects the antigen presenting cells by inhibiting their differentiation, antigen presentation, and production of co-stimulatory molecules and inflammatory cytokines, and promoting the expression of inhibitory molecules. Moreover, activation of VDR has been demonstrated to modulate the activity and cytokine production of different types of T cells by facilitating differentiation of regulatory T cells (T_reg_) and promoting a shift from T helper 1 (T_H_1) and T helper 17 (T_H_17) to T helper 2 (T_H_2) immune profiles [22,27,28,29]. In addition, there is evidence showing that VDR is upregulated in response to mitogen activation of cytotoxic T lymphocytes and B cells, suggesting a coordinated regulation of the VDR signaling pathway and response to stimuli [30,31,32]. The effects of vitamin D on the immune system are summarized in Figure 2. 

Besides its effects on calcium and phosphate homeostasis and immune function, vitamin D has been found to have biologic activities in multiple organ systems. To name a few, it plays a role in controlling glucose homeostasis by enhancing pancreatic β-cell function and improving insulin sensitivity [33,34]. It protects against vascular endothelial dysfunction by inducing nitric oxide production and maintaining endothelial stability [35,36]. It also has pro-differentiation and anti-proliferation effects on the keratinocytes and many types of cancer cells [37,38]. 

The broad range of theoretical health benefits of VDR activation has been supported by a multitude of studies that have showed associations between increased level of serum 25(OH)D concentration and decreased risks of infections, multiple chronic diseases, and all-cause mortality [39,40,41]. However, large-scale clinical trials have failed to demonstrate the benefit of supplementing vitamin D to the general population without known vitamin D deficiency in reducing risks of most chronic diseases, including diabetes, cardiovascular diseases, and cancer, except for a potential benefit in reducing the risks of autoimmune diseases [42,43,44,45]. While vitamin D supplementation is generally considered to raise the serum 25(OH)D level, increasing serum 25(OH)D does not necessarily reflect the increased level of circulating 1,25(OH)_2_D or increased VDR activation. This is based on the observation that circulating 1,25(OH)_2_D concentration can be normal or even high in individuals with vitamin D deficiency (defined by serum 25(OH)D < 20 ng/mL) due to the presence of secondary hyperparathyroidism that increases renal production of 1,25(OH)_2_D as a compensatory mechanism [19,46]. Therefore, the relationship between serum 25(OH)D, VDR activation, and health outcomes may be complex and requires further investigation. 

## 4. Vitamin D and COVID-19 Infection 

There are several proposed mechanisms by which vitamin D could reduce the risk and severity of COVID-19 infection. First, 1,25(OH)_2_D induces the macrophages and respiratory epithelial cells to produce antimicrobial peptides, including cathelicidin LL-37 that not only acts against invading bacteria and fungi by disrupting their cell membranes but also exhibits direct antiviral effects by altering viability of host target cells and destabilizing viral envelopes [47,48,49,50]. In addition, cathelicidins have been demonstrated to prevent lung injury secondary to oxygen toxicity [51]. This mechanism can be supported by the finding from a pilot study that showed increased serum cathelicidin in patients with sepsis who received a single enteral dose of 400,000 IUs of vitamin D_3_ compared to placebo [52]. Furthermore, a meta-analysis of 25 randomized controlled trials prior to the COVID era showed that vitamin D supplementation could reduce the risk of acute respiratory tract infection by approximately 12% compared to placebo [53]. 

The second mechanism involves the effects of VDR activation on the adaptive immune system. As discussed above, 1,25(OH)_2_D suppresses the activities of T_H_1 and T_H_17 and induces T_reg_ differentiation. This action results in decreased production of proinflammatory cytokines (i.e., IL-6, IL-8, IL-12, IL-17, TNF-α), which is thought to mitigate the cytokine storm seen in severe COVID-19 infection [22,27,28,29]. These actions of vitamin D are supported by clinical studies demonstrating improvement in inflammatory burden in autoimmune diseases mediated by T_H_1 and T_H_17 (i.e., rheumatoid arthritis, multiple sclerosis, psoriasis, and inflammatory bowel disease) [12,54,55,56,57]. 

Third, vitamin D plays a role in controlling the renin-angiotensin-aldosterone system [58,59]. Activation of VDR has been shown to inhibit expression of the angiotensin-converting enzyme 2 (ACE2) protein in the renal tubular cells, a receptor to which SARS-CoV-2 virus can bind to, and thus may protect against acute kidney injury caused by direct cytopathic effect of the virus [59,60]. 1,25(OH)_2_D also inhibits renin and angiotensin-converting enzyme and induces the expression of ACE2 in the lungs. This reduces the angiotensin II accumulation that is thought to mitigate the development of acute respiratory distress syndrome and cardiovascular complications in COVID-19 [61]. Decreased renin expression may also alleviate bradykinin storm [62]. 

Other proposed explanations include but are not limited to vitamin D’s pleiotropic actions which help stabilize vascular endothelium, prevent vascular thrombosis, and inhibit fibrotic changes in the lungs [35,63,64,65]. It is worth noting that DBP also has independent biological functions, including macrophage activation, fatty acid transport, chemotaxis, and actin scavenging [15]. These could possibly play a role in pathophysiology of COVID-19. Elevated serum F-actin concentration has been observed in patients with acute respiratory distress syndrome and is believed to be a causative factor of pulmonary vascular angiopathy [66,67]. DBP is thought to mitigate this process by working in the actin scavenging system with gelsolin to clear extracellular actin released by damaged cells from the circulation [68]. 

These proposed explanations have been supported by multitudes of studies showing an association between low levels of serum 25(OH)D and risk of COVID-19 infection and mortality even after controlling for potential confounders such as age, body mass index, and medical comorbidities [6,7,8,9,10,11]. On the other hand, the benefits of raising serum 25(OH)D by giving vitamin D supplementation to COVID-19 patients have been disputed. Although the impact of vitamin D supplementation in reducing COVID-19 associated mortality and other secondary morbidity outcomes was observable in meta-analyses, most individual clinical trials have failed to clearly demonstrate the benefit [69]. Of note, there is mounting evidence that the administration of 25(OH)D (calcifediol) may be of particular benefit in reducing the severity and inflammatory burden of COVID-19 [70,71,72,73,74]. This is possibly due to the pharmacokinetic difference that 25(OH)D is more bioavailable and able to raise serum 25(OH)D concentration faster than orally administered native vitamin D [74,75,76,77]. 

## 5. Genetic Variations of Vitamin D Metabolic Pathway and Various Clinical Outcomes 

Variation in the expression and activities of proteins involved in vitamin D metabolism can result in alterations in the kinetics and actions of vitamin D. Decreased enzymatic activity of the 7-dehydrocholesterol reductase, encoded by the *DHCR7* gene, results in accumulation of the substrate 7-dehydrocholesterol, thereby increasing endogenous production of vitamin D_3_ [78]. There are four major hepatic 25-hydroxylases encoded by the genes *CYP2R1*, *CYP27A1*, *CYP3A4*, and *CYP2J3*, which determine the rate of conversion of vitamin D to 25(OH)D [79]. Homozygous inactivating mutations of *CYP27B1* cause hereditary vitamin D-resistant rickets type 1 due to an inability to convert 25(OH)D into the active form 1,25(OH)_2_D. Homozygous inactivating mutations of *VDR* cause hereditary vitamin D-resistant rickets type 2 which manifests as rickets resistant to 1,25(OH)_2_D treatment and alopecia [80]. Loss-of-function mutations of *CYP24A1* lead to inability to catabolize 25(OH)D and 1,25(OH)_2_D that results in phenotypes of hypercalcemia and hypercalciuria that predispose affected individuals to kidney stones and nephrocalcinosis [81]. While loss-of-function mutations of *CYP27B1*, *CYP24A1*, and *VDR* have been shown to significantly affect calcium homeostasis. Homozygous inactivating mutation of *GC* causing complete deficiency of DBP has been reported in a woman born to consanguineous parents who had debilitating ankylosing spondylitis, low bone mass, and a normal calcium level [82].

Furthermore, certain genetic polymorphisms of these vitamin D-related genes mentioned above (*VDR*, *CYP2R1*, *CYP27B1*, *GC*, *CYP24A1, DHCR7*) have been shown to be associated with risks of several diseases in populational and functional studies. These diseases include but are not limited to osteoporosis, asthma, cardio-metabolic diseases, neurodegenerative diseases, autoimmune diseases, nonalcoholic fatty liver disease, type 1 diabetes, viral infections, pancreatitis, and several types of cancers [83,84,85,86,87,88,89,90,91,92,93,94,95,96,97]. 

The large volume of data supports the link between genetics of vitamin D metabolic pathway and non-skeletal outcomes. While it is inevitable that some of these findings are subject to false positive results secondary to multiple testing and/or post-hoc testing, it may implicate that there is inter-individual difference in responsiveness to vitamin D in the general population. This concept of individual responsiveness to vitamin D can be supported by a clinical trial of 71 patients with prediabetes receiving 3,200 IUs/day of vitamin D_3_ for 5 months that revealed robust changes in broad gene expression in approximately 50% of the patients [98]. This finding concurs with the observation from another study revealing that approximately 60% of healthy adults with baseline serum 25(OH)D <30 ng/mL who received 10,000 IUs per day of vitamin D_3_ for 6 months had robust response in genome-wide expression in peripheral blood mononuclear cells, compared to the other 40% with mild to moderate response despite comparable increases in the serum concentrations of 25(OH)D to the range of 60–90 ng/mL [99]. In addition, the subjects with robust genomic response to vitamin D displayed different patterns of serum metabolomic profiles compared with those with mild to moderate response [99,100]. The specific genomic or epigenomic factors that can explain this inter-individual difference are, however, currently unknown. 

## 6. Genetic Variations of Vitamin D Pathway and COVID-19 Infection 

A number of observational studies have evaluated the association between vitamin D-related polymorphisms and risk of COVID-19 infection and severity [101,102,103,104,105,106,107,108,109,110,111,112,113,114,115,116]. As shown in Table 1, most studies are from European and Middle eastern countries and included a relatively small number of participants. Six retrospective cohort studies from Turkey, Iran, Portugal, United Arab Emirates, Serbia, and Cuba investigated the association between vitamin D-related polymorphisms and COVID-19 severity [102,103,104,105,106,107]. Four case-control studies from Italy, Iran, and Cyprus reported the association between *VDR* polymorphism and COVID-19 susceptibility [108,109,110,111], while one study from Iran reported the association between *VDR* polymorphism and COVID-19 mortality [112].

As shown in Table 2, *VDR* polymorphisms including FokI (rs2228570, exon 2, C > T), TaqI (rs731236, exon 9, A > G) and BsmI (rs1544410, intron 8, G > A) were shown to be associated with the risk of COVID-19 and severity in more than one observational study [102,103,108,109,110,111,112]. However, the data from these studies are largely conflicting and inconclusive. A polymorphism of the *CYP2R1* gene (rs10741657, 5′UTR, G > A) was found to influence the severity of COVID-19 in two studies in the United Arab Emirates and Serbia [105,106]. An ecological study by Batur et al. [101] demonstrated that the prevalence of GT and TT genotypes of a polymorphism of the *GC* gene (rs7041, or BsuRI, exon 11, G > T) in each of the ten countries was associated with increased prevalence and decreased mortality rates among these countries. However, this finding was not confirmed in an observational study by Jafarpoor et al. [109]. Many other polymorphisms of the intronic and untranslated regions of the genes *DHCR7*, *GC*, and *VDR* were found to be associated with increased risk of critical COVID-19 disease in a study by Al-Anouti et al. [105]. However, in this study, a high throughput method was used for genotyping and the analysis was not adjusted for multiple testing; therefore, false positive results were very likely. 

Taken together, there is slight evidence that polymorphisms of the *VDR* and *GC* genes may influence the risks of infectivity and severity of COVID-19, although the confidence of these findings is low due to the discrepancy in the results across the studies and the concern for false positivity in genomic research. Additional well-powered studies (such as genome-wide association studies) are needed to further elucidate the potential association between genetic variations in the vitamin D metabolic pathway and COVID-19.

## 7. Mendelian Randomization Studies of Vitamin D and COVID-19

Many Mendelian randomization studies utilized data from the UK biobank and the COVID-19 Host Genetics Initiative and generated models to predict serum 25(OH)D level or vitamin D deficiency (Table 1) [113,114,115,116]. These studies did not find any association between genetically predicted serum 25(OH)D level and COVID-19 risk. The models included variants of the genes *CYP2R1*, *CYP24A1*, *DHCR7*, and *GC* among several other SNPs [113,114,115,116]. While these studies point against the benefit of raising serum 25(OH)D level for COVID-19 risk and severity, the findings should be interpreted with caution as these models that included only genetic variants were not associated with unmeasured variables. It is known that environmental factors (i.e., vitamin D intake and UVB exposure) are also major determinants of vitamin D status in addition to genetics [117,118,119]. 

## 8. Functional Studies of Genetic Variations of the *VDR* and *GC* Genes

There are a few experimental studies that demonstrated functional significance of genetic variations of the *VDR* and *GC* genes. For instance, the FokI polymorphism results in VDR proteins with different structures: long f-VDR and short F-VDR. It has been well demonstrated in a transfection experimental study by van Etten et al. [120] that the shorter F-VDR resulted in higher NF-kB and NFAT-driven transcription and higher IL-12p40 promoter-driven transcription. Furthermore, homozygous short FF VDR genotype, compared with long ff VDR genotype, results in higher mRNA and protein expression of IL-12 in the macrophages and dendritic cells and stronger phytohemagglutinin-induced lymphocytic proliferation [120]. The CDX2 site, located in the promoter region of the VDR, is a binding site of the transcription factor caudal type homeobox-2 (CDX-2). CDX2 polymorphism affects the binding affinity of the CDX-2 transcription factor, *VDR* promoter methylation and thus *VDR* mRNA expression [121,122]. In an ex vivo study of peripheral blood mononuclear cell cultures of 51 patients with pulmonary tuberculosis and 60 healthy subjects, the CDX2 GG genotype and the combination baT haplotype of the 3′ untranslated region SNPs Apa I, Bsm I, and Taq I were associated with decreased T_H_1 response and increased IL-10 in response to culture filtrate antigen and 1,25(OH)_2_D [123]. 

Variations of the *GC* gene result in different isoforms of the DBP distinguished by electrophoresis. Among over 120 isoforms, there are three major isoforms that have been widely studied, namely Gc1f (rs7041 T and rs4588 C alleles), Gc1s (rs7041 G and rs4588 C alleles and Gc2 (rs7041 T and rs4588 A alleles). The Gc1s isoform has a higher affinity for 25(OH)D than the Gc1f isoform, with the Gc2 allele in between [15]. In addition, the Gc1f isoform carries the highest ability to be converted into the macrophage activating factor (MAF) by the sequential reactions of β-galactosidase and sialidase by T and B cells, respectively [124]. This explains the functional significance of these major *GC* polymorphisms (rs7041 and rs4588), which are shown to be associated with various clinical outcomes, including the risk of COVID-19. 

## 9. Conclusions

The immunomodulatory and metabolic effects of vitamin D and VDR activation have been considered to have therapeutic and preventive potential in COVID-19 infection. Furthermore, DBP, encoded by the *GC* gene, is known to have independent immunological and actin-scavenging effects that may affect inflammation in COVID-19. Observational studies have shown that serum 25(OH)D concentration is inversely associated with the risk and severity of COVID-19. However, evidence from randomized clinical trials of vitamin D supplementation is weaker. This may suggest that raising serum 25(OH)D may not completely reflect increased activation of the intracellular VDR in the widespread tissues. 

Another important aspect of the vitamin D-COVID-19 theory is that common polymorphisms in the *VDR* and *GC* genes have been shown to influence the susceptibility and severity of COVID-19. Although the strength of evidence is limited by a small number of studies and participants as well as possible false positivity, there is biological plausibility based on experimental studies that certain polymorphisms of the *VDR* and *GC* genes can affect immune function.

Therefore, it is possible that inter-individual differences in the function of *VDR* and *GC* based on their genetic variations may have confounded the results of the clinical trials that aimed to determine the impact vitamin D supplementation on COVID-19 and other clinical outcomes. Further investigations are warranted to address the potential role of VDR activation and DBP in pathophysiology of COVID-19 considering the genetic variations of the vitamin D metabolic pathway. In particular, *VDR* and *GC* polymorphisms should be taken into account as potential effect modifiers in studies aiming to determine causality of vitamin D and COVID-19-related outcomes. 

## Figures and Tables

**Table 1:** Characteristics of studies investigating the association between vitamin D-related genetic variations and COVID-19 susceptibility and severity.

Study	Country	Study Design	Population Characteristics/Methods	Studied Vitamin D-Related Gene(s)	Outcomes
Batur et al., 2021 [101]	Multiple countries	Ecological study	Allele frequencies were obtained from data reported in five cohort and two systematic review and meta-analysis studies. Number of cases of COVID-19 per million population was obtained from the WHO COVID-19 Situation Report—164 in each of the ten countries (China, Japan, Nigeria, Kenya, Mexico, Italy, Turkey, Finland, Germany, Czech).	*GC*	- Prevalence per million of COVID-19 in each country - Mortality rates per million of COVID-19 in each country
Apaydin et al., 2021 [102]	Turkey	Retrospective cohort	297 with RT-PCR confirmed COVID-19 admitted to Marmara University Education and Research Hospital between April and October 2020	*VDR*	- COVID-19 disease severity - Intensive care unit admission - Mortality
Abdollahzadeh et al., 2021 [103]	Iran	Retrospective cohort	500 COVID-19 patients hospitalized at different hospitals in Iran between 5 May and 25 September, 2020.	*VDR*	- Signs and symptoms of COVID-19
Freitas et al., 2021 [104]	Portugal	Retrospective cohort	491 patients with laboratory confirmed COVID-19 from Santa Maria hospital and São João hospital	*CYP2R1*, *CYP24A1*, *DHCR7*, *GC* and *VDR*	- COVID-19 disease severity
Al-Anouti et al., 2021 [105]	United Arab Emirates	Retrospective cohort	646 patients with RT-PCT confirmed COVID-19 infection from the Sheikh Khalifa Medical City, quarantine area in Abu Dhabi and Rashid Hospital in Dubai between April 2020 and January 2021	*CYP2R1*, *GC* and *VDR*	- Critical illness requiring hospital admission organ support (i.e., hypoxia, respiratory failure, septic shock or multiorgan failure)
Kotur et al., 2021 [106]	Serbia	Retrospective cohort	120 adult and pediatric patients with COVID-19 treated at the Clinic of Pulmonology, Clinical Center of Serbia and Children’s Hospital for Lung Diseases and Tuberculosis, Medical Center “Dr Dragiša Mišovic,” Belgrade, Serbia, between April and June of 2020.	*DHCR7*, *CYP2R1*, *GC*, *VDR*	- COVID-19 disease severity
Peralta et al., 2021 [107]	Cuba	Retrospective cohort	104 patients with COVID-19 randomly recruited from Cuban citizens aged >1 year old	*VDR*	- COVID-19 disease severity
Balzanelli et al., 2022 [108]	Italy	Case-control	41 patients with COVID-19 and 43 healthy controls recruited from the 118 Pre-hospital and Emergency Department of SG Moscati Hospital of Taranto, Italy between September 2020 and October 2020.	*VDR*	- COVID-19 susceptibility
Jafarpoor et al., 2022 [109]	Iran	Case-control	188 hospitalized patients with COVID-19 and 218 patients with suspected COVID-19 with mild signs recruited from hospitals affiliated with the Iran University of Medical Sciences between March 2020 and June 2020	*VDR* and *GC*	- COVID-19 hospitalization/susceptibility
Mamurova et al., 2022 (preprint) [110]	Cyprus	Case-control	600 patients admitted to Near East University Hospital consisting of 100 with Alpha variant, 100 with Delta variant, 100 with Omicron variant and 300 with negative COVID-19 RT-PCR test	*VDR*	- COVID-19 susceptibility
Zeidan et al., 2022 [111]	Egypt	Case-control	180 patients with COVID-19 and 200 age-, sex-, season-at-enrollment-matched controls recruited from Cairo, Ain-Shams, and Assuit University hospitals between October 2020 and March 2021	*VDR*	- COVID-19 susceptibility
Albu-Mohammed et al., 2022 [112]	Iran	Case-control	1734 patients recovered patients with COVID-19 and 1450 deceased patients with COVID-19 referred to the Ilam University of Medical Sciences between November 2020 and February 2022	*VDR*	- COVID-19 mortality
Butler-Laporte et al., 2020 [113]	The United Kingdom	Mendelian randomization study	Genetic variants associated with 25(OH)D levels in a GWAS of 443,734 participants of European ancestry including 401,460 from the UK Biobank were used as instrumental variable. GWASs of COVID-19 susceptibility, hospitalization, and severe disease from the COVID-19 Host Genetics Initiative were used as outcome GWASs.	*CYP2R1, CYP24A1, DHCR7* and *GC* among other genes in a model predicting serum 25(OH)D level	- COVID-19 susceptibility - Hospitalization - COVID-19 disease severity
Patchen et al., 2021 [114]	The United Kingdom	Mendelian randomization study	Data from genome-wide analyses in the population-based UK Biobank and SUNLIGHT Consortium were used as instrumental variable. Data from the COVID-19 Host Genetics Initiative were used as outcome GWASs. Participants included 17,965 COVID-19 cases including 11,085 laboratory or physician-confirmed cases, 7885 hospitalized cases and 4336 severe respiratory cases and 1,370,547 controls, primarily of European ancestry.	*CYP2R1*, *CYP24A1*, *DHCR7* and *GC* among other genes in a model predicting serum 25(OH)D level	- COVID-19 susceptibility - Hospitalization - COVID-19 disease severity - Severe respiratory COVID-19
Amin et al., 2022 [115]	The United Kingdom	Mendelian randomization study	Data from a GWAS in the population-based UK Biobank were used as instrumental variable. Data from the COVID-19 Host Genetics Initiative were used as outcome GWASs.	*CYP2R1*, *CYP24A1*, *DHCR7* among *GC* among other genes in a model predicting vitamin D deficiency	- COVID-19 susceptibility - COVID-19 severity
Cui et al., 2022 [116]	The United Kingdom	Mendelian randomization study	Data from genome-wide analyses in the population-based UK Biobank and SUNLIGHT Consortium were used as instrumental variable. Data from the COVID-19 Host Genetics Initiative were used as outcome GWASs.	Genes in a model predicting serum 25(OH)D level	- COVID-19 susceptibility - Hospitalization - COVID-19 disease severity

Abbreviations: 25(OH)D: 25-hydroxyvitamin D; COVID-19: Coronavirus disease 2019; GWAS: Genome-wide association study; RT-PCR: Reverse transcription polymerase chain reaction; UK: The United Kingdom.

**Table 2:** Association between vitamin D-related genetic variations and COVID-19 susceptibility and severity.

Gene	SNP	Location	Allele	Finding(s)
*DHCR7*	rs12785878	Intron 2	T > G	- Kotur et al., 2021 [106]: TT genotype associated with mild-moderate COVID-19 vs. severe COVID-19 (*p* = 0.03).
	rs4944979	Intron 16	G > T	- Al-Anouti et al., 2021 [105]: GG genotype associated with decreased risk of critical COVID-19 (*p* = 0.02).
	rs4944997	Intron 18	G > A	- Al-Anouti et al., 2021 [105]: GG genotype associated with decreased risk of critical COVID-19 (*p* = 0.02).
	rs4944998	Intron 18	G > C	- Al-Anouti et al., 2021 [105]: GG genotype associated with decreased risk of critical COVID-19 (*p* = 0.02).
	rs4944076	Intron 20	A > G	- Al-Anouti et al., 2021 [105]: AA genotype associated with decreased risk of critical COVID-19 (*p* = 0.008).
	rs10898210	Intron 20	A > G	- Al-Anouti et al., 2021 [105]: AA genotype associated with decreased risk of critical COVID-19 (*p* = 0.009).
*CYP2R1*	rs10741657	5′UTR	G > A	- Al-Anouti et al., 2021 [105]: AA genotype associated with decreased risk of critical COVID-19 (*p* = 0.004). - Kotur et al., 2021 [106]: GG genotype associated with increased risk of severe COVID-19.
*GC*	rs4588	Exon 11	C > A	- Batur et al., 2021 [101]: no association with prevalence and mortality rates
	rs7041 (BsuRI)	Exon 11	G > T	- Batur et al., 2021 [101]: GT genotype associated with increased prevalence and mortality rates compared with TT genotype (*p* < 0.05). - Jafarpoor et al., 2022 [109]: no association with likelihood of COVID-19.
	rs113876500	Upstream of Exon 1	G > T	- Al-Anouti et al., 2021 [101]: AA genotype associated with decreased risk of critical COVID-19 (*p* = 0.02).
	rs59241277	Intron 1	A > G	- Al-Anouti et al., 2021 [101]: AA genotype associated with decreased risk of critical COVID-19 (*p* = 0.005).
	rs182901986	Intron 1	G > A	- Al-Anouti et al., 2021 [101]: GG genotype associated with decreased risk of critical COVID-19 (*p* = 0.01).
	rs113574864	Intron 6	C > T	- Al-Anouti et al., 2021 [101]: CC genotype associated with decreased risk of critical COVID-19 (*p* = 0.005).
	rs60349934	Intron 6	T > C	- Al-Anouti et al., 2021 [101]: TT genotype associated with decreased risk of critical COVID-19 (*p* = 0.01).
	rs2282679	Intron 12	T > G	- Freitas et al., 2021 [104]: associated with severity (*p* = 0.005). - Kotur et al., 2021 [106]: no association with COVID-19 severity.
*VDR*	rs11568820 (CDX2)	Promotor	G > A	- Abdollahzadeh et al., 2021 [103]: C allele associated with decreased COVID-19 severity compared with c allele (*p* < 0.05).
	rs4516035 (EcoRV)	Promotor	T > C	- Abdollahzadeh et al., 2021 [103]: e allele associated symptomatic COVID-19 compared with E allele (*p* < 0.05).
	rs2228570 (FokI)	Exon 2	C > T	- Apaydin et al., 2021 [102]: Ff (CT) genotype associated with increased COVID-19 severity. - Abdollahzadeh et al., 2021 [103]: f allele associated with symptomatic COVID-19 compared with f allele (*p* < 0.05). - Kotur et al., 2021 [106]: no association with COVID-19 severity. - Balzanelli et al., 2022 [108]: ff (TT) genotype associated with decreased COVID-19 susceptibility (*p* < 0.05). - Jafarpoor et al., 2022 [109]: f (T) allele associated with increased likelihood of COVID-19 (*p* = 0.001) - Zeiden et al., 2022 [111]: FF (CC) genotype associated with increased likelihood of COVID-19 compared with ff (TT) genotype (*p* < 0.001). - Mamuruva et al., 2022 (preprint) [110]: f (T) allele associated with increased likelihood of COVID-19.
	rs731236 (TaqI)	Exon 9	A > G	- Apaydin et al., 2021 [102]: TT genotype associated with ICU admission compared with Tt genotype (*p* = 0.08). - Abdollahzadeh et al., 2021 [103]: no association with COVID-19 severity. - Peralta et al. 2021 [107]: no association with symptomaticity of COVID-19 - Balzanelli et al., 2022 [108]: tt genotype associated with decreased COVID-19 susceptibility (*p* < 0.05). - Jafarpoor et al., 2022 [109]: no association with likelihood of COVID-19. - Mamuruva et al., 2022 (preprint) [110]: t allele associated with increased likelihood of COVID-19. - Albu-Mohammed et al., 2022 [112]: Tt and tt genotype associated with increased COVID-19 Alpha variant mortality; tt genotype associated with increased COVID-19 Delta variant mortality; no association with COVID-19 Omicron BA.5 variant mortality
	rs757343 (Tru9I)	Exon 9	A > G	- Abdollahzadeh et al., 2021 [103]: u allele associated with increased COVID-19 severity compared with U allele (*p* < 0.05).
	rs11574018	Intron 1	T > C	- Al-Anouti et al., 2021 [101]: TT genotype associated with decreased risk of critical COVID-19 (*p* = 0.04).
	rs11574024	Intron 1	G > T	- Al-Anouti et al., 2021 [101]: GG genotype associated with decreased risk of critical COVID-19 (*p* = 0.04).
	rs1544410 (BsmI)	Intron 8	G > A	- Apaydin et al., 2021 [102]: no association with COVID-19 severity. - Abdollahzadeh et al., 2021 [103]: b allele associated with increased COVID-19 severity compared with B allele (*p* < 0.05). - Balzanelli et al., 2022 [108]: BB genotype associated with slightly increased COVID-19 susceptibility (*p* < 0.05).
	rs7975232 (ApaI)	Intron 8	C > A	- Apaydin et al., 2021 [102]: aa genotype associated with COVID-19 mortality (*p* = 0.001) - Abdollahzadeh et al., 2021 [103]: no association with COVID-19 severity. - Jafarpoor et al., 2022 [109]: no association with likelihood of COVID-19.
	rs739837 (BglI)	3′UTR	G > T	- Abdollahzadeh et al., 2021 [103]: no association with COVID-19 severity. - Albu-Mohammed et al., 2022 [112]: Gg and gg genotypes associated with increased COVID-19 Omicron BA.5 variant mortality; no association with COVID-19 Alpha and Delta variant mortality.
